# Construction and analysis of a lncRNA-miRNA-mRNA network based on competitive endogenous RNA reveal functional lncRNAs in oral cancer

**DOI:** 10.1186/s12920-020-00741-w

**Published:** 2020-06-22

**Authors:** Junhao Yin, Xiaoli Zeng, Zexin Ai, Miao Yu, Yang’ou Wu, Shengjiao Li

**Affiliations:** 1 Department of Oral and Maxillofacial Surgery, School and Hospital of Stomatology, Tongji University, Shanghai, 200072 China; 2 Shanghai Engineering Research Center of Tooth Restoration and Regeneration, Shanghai, 200072 China

**Keywords:** Competitive endogenous RNA, Oral cancer, Long non-coding RNA, HCG22

## Abstract

**Background:**

A growing evidence suggests that long non-coding RNAs (lncRNAs) can function as a microRNA (miRNA) sponge in various diseases including oral cancer. However, the pathophysiological function of lncRNAs remains unclear.

**Methods:**

Based on the competitive endogenous RNA (ceRNA) theory, we constructed a lncRNA-miRNA-mRNA network in oral cancer with the human expression profiles GSE74530 from the Gene Expression Omnibus (GEO) database. We used topological analysis to determine the hub lncRNAs in the regulatory ceRNA network. Then, function enrichment analysis was performed using the clusterProfiler R package. Clinical information was downloaded from The Cancer Genome Atlas (TCGA) database and survival analysis was performed with Kaplan-Meier analysis.

**Results:**

A total of 238 potential co-dysregulated competing triples were obtained in the lncRNA-associated ceRNA network in oral cancer, which consisted of 10 lncRNA nodes, 41 miRNA nodes and 122 mRNA nodes. Additionally, we found lncRNA HCG22 exhibiting superior potential as a diagnostic and prognostic marker of oral cancer.

**Conclusions:**

Our findings provide novel insights to understand the ceRNA regulation in oral cancer and identify a novel lncRNA as a potential molecular biomarker.

## Background

Oral cancer is a malignant neoplasia with low overall survival rates [1], among which oral squamous cell carcinoma (OSCC) is the most common type [2]. The predilection sites of oral cancer include the buccal mucosa, tongue and lower lip, and the occurrence is higher in people over fifty [3]. In 2018, the worldwide age-standardized rate per 100,000 person-years of oral cancer was 4.0, and 64.2% of the global incidence clustered in South Asia according to the Global Cancer Observatory [4]. It is well established that the major risk factors for oral cancer include tobacco chewing, smoking, alcohol drinking, excessive sunlight exposure and Human Papilloma Virus (HPV). In addition, several genetic factors such as tumor suppressor genes, oncogenes and regulatory genes may also play a crucial role in oral carcinogenesis [5, 6]. Genetic alterations to the genes of TP53, NOTCH1 and PIK3CA affect the epithelial cells and contribute to the microenvironment alterations such as the ROS accumulation, overproduction of cytokines and epithelial to mesenchymal transition, inducing uncontrolled cell proliferation, growth and tumorigenesis [7, 8].

Initially, it was thought that coding RNAs play the most essential roles in cancer, while non-coding RNAs (ncRNAs) are no more than transcriptional noise. However, there is an increasing evidence indicating the important regulatory roles of ncRNAs in the occurrence and progression of various cancer types [9]. NcRNAs include circular RNAs, microRNAs, intronic RNAs and long non-coding RNAs [10]. MicroRNAs (miRNAs) are representative ncRNAs of 18–25 nucleotides in length [11]. They regulate the expression of target genes by inhibiting their translation and accelerating their degradation [12], and have been shown to be involved in many different physiological and pathological processes, including the epithelial-mesenchymal transition, metabolism, survival and more. First confirmed in 2005, long non-coding RNAs (lncRNAs) represent another type of ncRNA and also lack any protein-coding capacity [13]. The nucleotide sequence length of lncRNAs is typically between 200 and 10,0000 nt. In addition, some lncRNAs may be associated with cancer phenotypes [14] and were chosen as new diagnostic and prognostic biomarkers for various cancer types, including nasopharyngeal carcinoma [15], gastric cancer [16] and prostate cancer [17]. However, the role of lncRNAs in the development of OSCC remains to be explored.

The competitive endogenous RNA (ceRNA) hypothesis [18], a novel regulatory mechanism that received attention in 2011, indicated that circular RNAs, lncRNAs and pseudogenes can regulate the abundance of miRNAs as molecular sponges. The theory may lead to essential clues to understand gene regulatory networks in many diseases, including OSCC. For example, one of the initial findings indicate that the pseudogene PTENP1 has the miRNA sponge capacity to regulate the levels of the PTEN gene in cancer [19]. ANRIL is a miRNA sponge for miR-125a-3p that regulates the abundance of FGFR1 to promote the tumorigenesis of head and neck squamous cell carcinoma [20]. Kanagaraj Arun et al. [21] found that both lncRNAs PTENP1-AS and GAS5 could act as tumor suppressive ceRNAs in gastric cancer. These studies have shown lncRNAs to be potential biomarkers for the diagnosis and prognosis of various diseases.

In this study, we performed an analysis of the RNA expression profiles in oral cancer patients from the Gene Expression Omnibus (GEO) database at the National Center for Biotechnology Information (NCBI) to screen the differentially expressed lncRNAs (DELs) and mRNAs (DEMs) that are related to oral cancer. Then, we constructed a lncRNA-associated ceRNA network by combining bioinformatics and correlation analyses to find the hub lncRNAs in OSCC. Meanwhile, sequence data and clinical information were obtained from The Cancer Genome Atlas (TCGA) database. To further investigate the relationship between the expression pattern and clinical information of the oral cancer samples, the Kaplan-Meier survival analysis of hub lncRNAs was carried out.

## Methods

### GEO data collection

We retrieved the human expression profiles (accession number: GSE74530) of oral cancer from NCBI GEO [22], which were extracted from a study carried out by Oghumu et al. [23]. The expression data included the lncRNA and mRNA expression profiles. The samples were derived from the tumor tissue and adjacent non-tumor tissue of six OSCC patients who were enrolled in a Phase 0 clinical trial study. The microarray platform used to analyze these data was the GPL570 Affymetrix Human Genome U133 Plus 2.0 Array and the direct web link was listed in Additional file 1.

### Data quality assessment

The AffyPLM package [24] in the R statistical language was used to analyze the data quality at the probe level. The boxplot representations of residuals and weights from probe level fits were obtained, by which the trend consistency of the expression data can be tested. The degradation of the RNA was assessed using the AffyRNAdeg function of AffyPLM, to assure the consistent trend and RNA integrity of the microarray dataset before further processing.

### Data pre-processing and screening of differentially expressed lncRNAs and mRNAs

In order to identify the biological significance of each probe, the comprehensive gene annotation files were obtained from GENCODE in GTF format. The GENCODE annotation was the default gene annotation displayed in the Ensembl Genome Browser. The transcripts with a length of more than 200 nucleotides and a biotype categorized as “non_coding”,“processed_transcript”,“lincRNA”,“retained_intron”,“antisense”,“sense_overlapping”, “sense_intronic” and “bidirectional_promoter_lncrna” were labeled as “lncRNAs”, while the transcripts with a biotype categorized as “protein_coding” were labeled as “mRNAs”. Finally, 1210 expressed lncRNAs and 16,434 expressed mRNAs were annotated.

The Affymetrix probe level data were obtained by reading the CEL files using the ReadAffy function of the Affy R package [25]**,** and then the raw data were preprocessed (background correction, normalization and summary expression computation). We used the limma Bioconductor package [26] to explore the differentially expressed genes (DEGs) between adjacent normal tissue and tumors groups, including lncRNAs (DELs) and mRNAs (DEMs). The DELs and DEMs were filtered according to the cut-off criteria of adjusted *p*-value < 0.05 and |log_2_ (fold change) | > 1.

### Prediction of target miRNAs of DELs and miRNA-target interactions

The lncRNA targets of the miRNAs were collected using the transcriptome-wide mircoRNA target predictions from the miRcode database [27], which contained more than 10,000 lncRNAs. The predictions were made based on the GENCODE transcripts. Interactions between miRNA and mRNA were found in the 3 miRNA databases of miRTarBase 6.0 [28]**,** miRDB [29] and TargetScan 7.0 [30], with the criteria that each target mRNA appears in at least 2 of them. The mRNA targets of the miRNA-mRNA interactions were merged with DEMs for further analysis.

### Construction of the lncRNA-associated ceRNA network and topological analysis

A lncRNA-miRNA-mRNA interaction was identified as a potential ceRNA triple based on the following criteria [31]:

(1) The Pearson’s correlation coefficients (PCCs) between each DELs-DEMs pair in oral cancer were calculated. The DEL-DEM pairs were regarded as co-dysregulated DEL-DEM pairs if the thresholds of the PCC value ranked in the top 0.05 percentile (PCC > 0.886) with a *p*-value < 0.05.

(2) Once it was confirmed that both mRNA and lncRNA in a co-dysregulated DEL-DEM pair were targeted by the same miRNA, this lncRNA-miRNA-mRNA interaction would then be identified as a potential co-dysregulated competing triple.

To give an insight into the roles of lncRNAs in the ceRNA network, we assembled all the potential co-dysregulated competing triples to build the lncRNA-miRNA-mRNA network and visualized the regulation network built from these interactions using the Cytoscape 3.7.1 [32] software.

Topological analysis is important to discover information in complex data sets. In order to study the geometric relationships between the data nodes, we computed the node degree and betweenness centrality (BC) of each node, which are both network topological features. Then, the nodes with a high node degree (> 5 connections) and a greater BC value were considered to be the hub nodes in the regulation network, which were more likely to play an important role in oral cancer [33].

### Functional enrichment analysis

In order to explore the functions of the obtained lncRNAs, we performed Gene Ontology (GO) enrichment analysis and Kyoto Encyclopedia of Genes and Genomes (KEGG) pathway analysis of mRNAs in the lncRNA-associated regulation network using the clusterProfiler R package [34]. Simultaneously, the GO interaction network was built using the Biological Networks Gene Ontology tool (BiNGO) in the Cytoscape software [32]. We set *p*-value < 0.05 and Benjamini-Hochberg corrected *p*-value < 0.05 as the thresholds of the functional categories.

### Construction of the key lncRNA-associated subnetworks

The lncRNAs with high node degree and BC value were chosen as hub lncRNAs, which were used with their related miRNAs and mRNAs in the regulation network to construct the subnetworks using the Cytoscape software. After that, gene functional enrichment analyses were performed for each subnetwork.

### TCGA data collection

The RNAseq data and corresponding clinical data of 319 oral cancer samples (including 19 buccal mucosa samples, 54 floor of mouth samples, 116 larynx samples, and 130 tongue samples from the TCGA-HNSC project) were downloaded from The Cancer Genome Atlas (TCGA) database (http://portal.gdc.cancer.gov/; dbGaP Study Accession:phs000178) using the TCGAbiolinks R package [35]. The expression data and clinical information included 319 oral cancer samples and 44 matched normal samples. Meanwhile, 14 samples of non-solid tumors were excluded from the dataset and the accession numbers of all samples were included in Additional file 2. The raw data were preprocessed by TCGAanalyze_Preprocessing and TCGAanalyze_purity function of TCGAbiolinks. A total of 236 oral cancer samples and 44 normal samples were filtered.

### Differentially expressed analysis and survival analysis

The TCGAbiolinks R package was used to filter the differentially expressed genes between normal and tumor samples according to the cut-off criteria of adjusted *p*-value < 0.05 and |log2 (fold change) | > 1. GO function analysis was carried out for up-regulated and down-regulated genes respectively. Furthermore, to determine the prognosis of oral cancer patients in relation to differentially expressed RNA signatures, survival curves of hub IncRNAs were analyzed using the survival package in the R statistical language with a threshold of log-rank *P* < 0.05.

## Results

### Data quality assessment and preprocessing

**Fig. 1:**
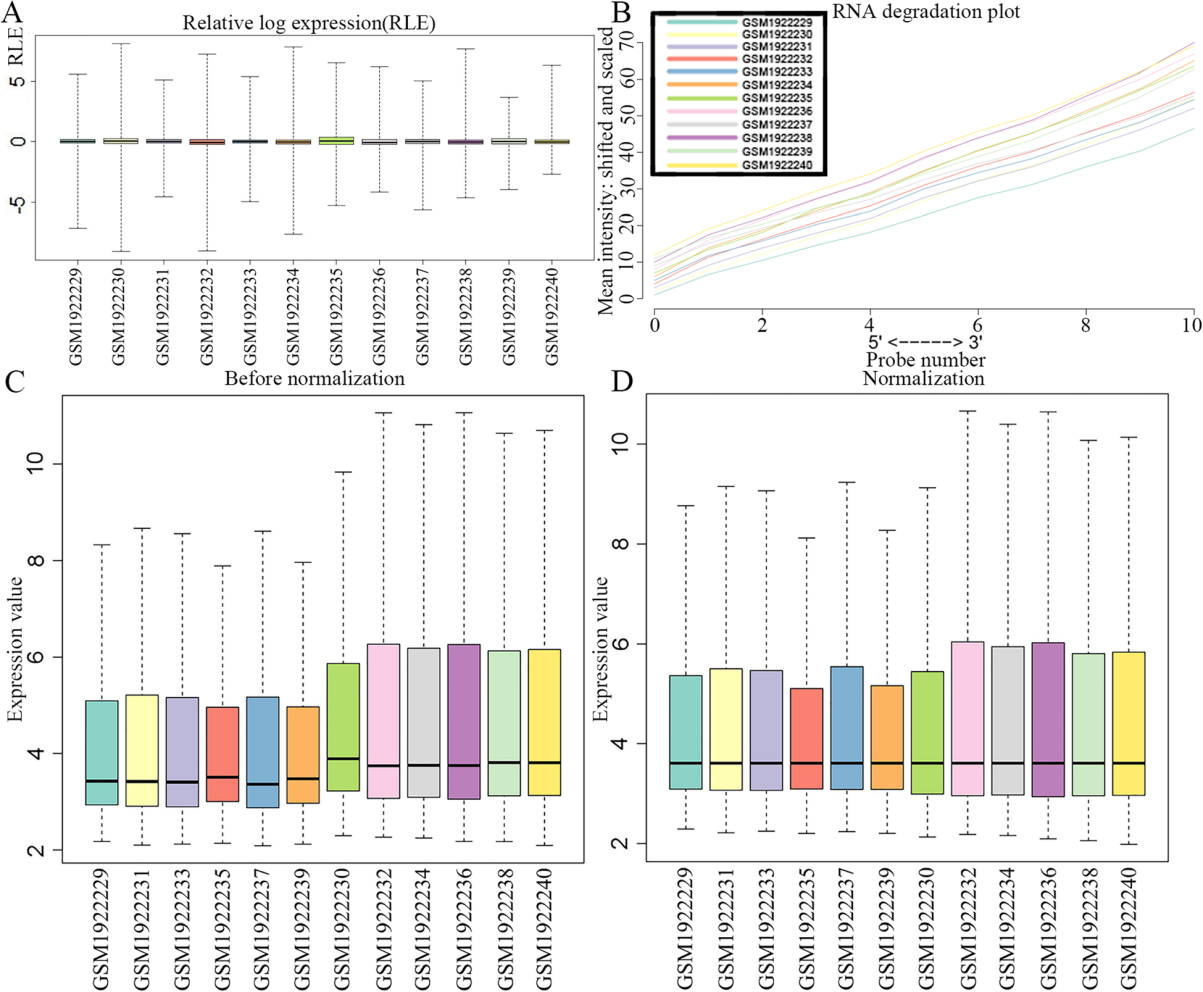
Data quality assessment. **a** Boxplot representation of the relative log expression (RLE). **b** RNA degradation plot, twelve curves represent twelve different samples, respectively. **c** Boxplot of intensity distributions in the raw data. **d** Boxplot of intensity distributions in the normalized data. The relative expression values are comparable among all twelve samples after normalization

Regression analysis of the raw data was performed using the affyPLM R package. The relative log expression (RLE) plot revealed that the gene expression levels in GSE74530 were consistent with the median approaching 0 (Fig. 1a), indicating that the quality of the expression data was reliable. As for the RNA degradation plot, it showed that the RNA integrity has a good quality (Fig. 1b), and all the 12 samples can be used for further analysis. A total of 16,434 mRNAs and 1210 lncRNAs were identified in the microarray data using the human comprehensive gene annotation from GENCODE. As shown in Fig. 1c, there were no obvious outliers in the spread and location in boxplot, and small discrepancies can be sufficiently removed by normalization. The median values of 12 samples were almost at the same level after normalization (Fig. 1d**)**, which effectively corrected the systematic differences between the chips.

### The screening results of DELs and DEMs

**Fig. 2:**
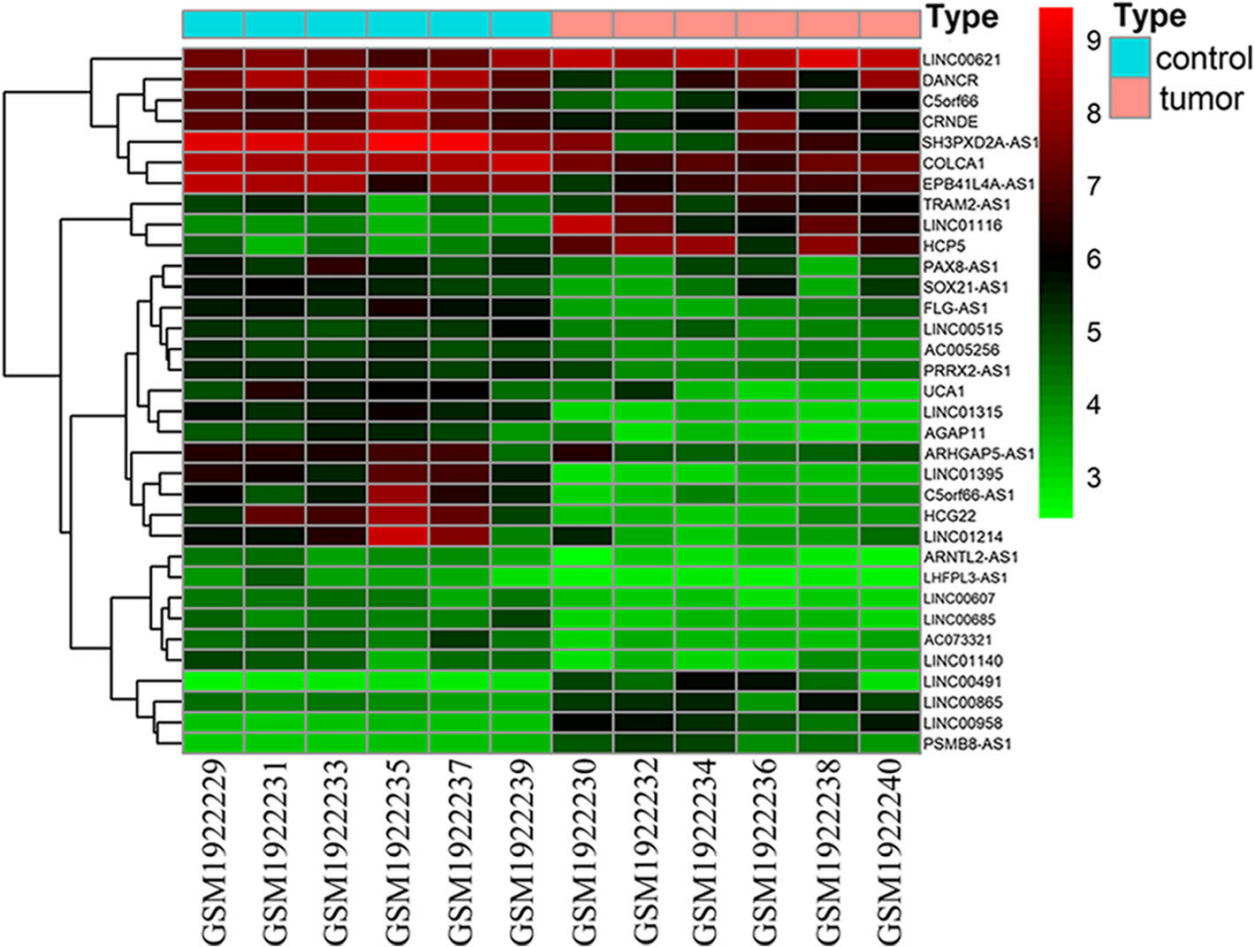
Heatmap of differentially expressed lncRNAs in oral cancer. The horizontal axis shows the names of twelve samples. The vertical axis presents the gene names

**Fig. 3:**
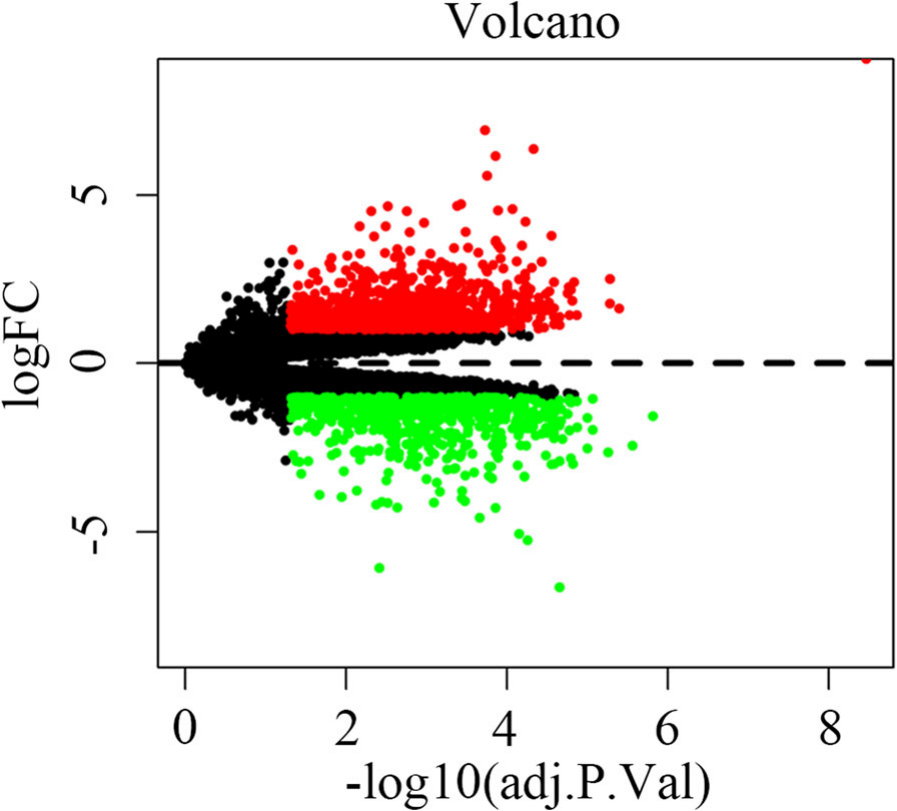
Volcano plot of all differentially expressed genes in oral cancer. FC are fold-change. Downregulated genes are green and upregulated genes are red

We identified 34 DELs and 1641 DEMs by comparing the tumor groups with the adjacent normal tissue group using the limma package. The expression of these DELs and all the DEGs are visualized in a heatmap (Fig. 2) and a volcano plot (Fig .3), respectively. The miRNA targets of the lncRNAs were predicted using the miRcode database in R. The target mRNAs of these miRNAs were obtained using the three highly reliable microRNA target prediction databases of miRTarBase, TargetScan and miRDB, and the result was intersected with the above-mentioned DEMs.

As a result, we got a total of 2137 reliable miRNA-mRNA pairs and 162 predicted lncRNA-miRNA pairs (including 11 lncRNAs, 51 miRNAs and 851 mRNAs), which were used for further analysis.

### Construction of the ceRNA network in oral cancer

In order to reduce the false positives, we calculated the PCC between the 34 DELs and 1641 DEMs, and the co-expressed pairs with the top 5% PCC value (PCC > 0.886) were defined as the significant co-dysregulated competing pairs. Next, the results were merged with the 11 lncRNAs, 51 miRNAs and 851 mRNAs from the previous screening step. The intersection resulted in the selection of a total of 238 potential co-dysregulated competing triples, the full list is shown in Additional file 3.

**Fig. 4:**
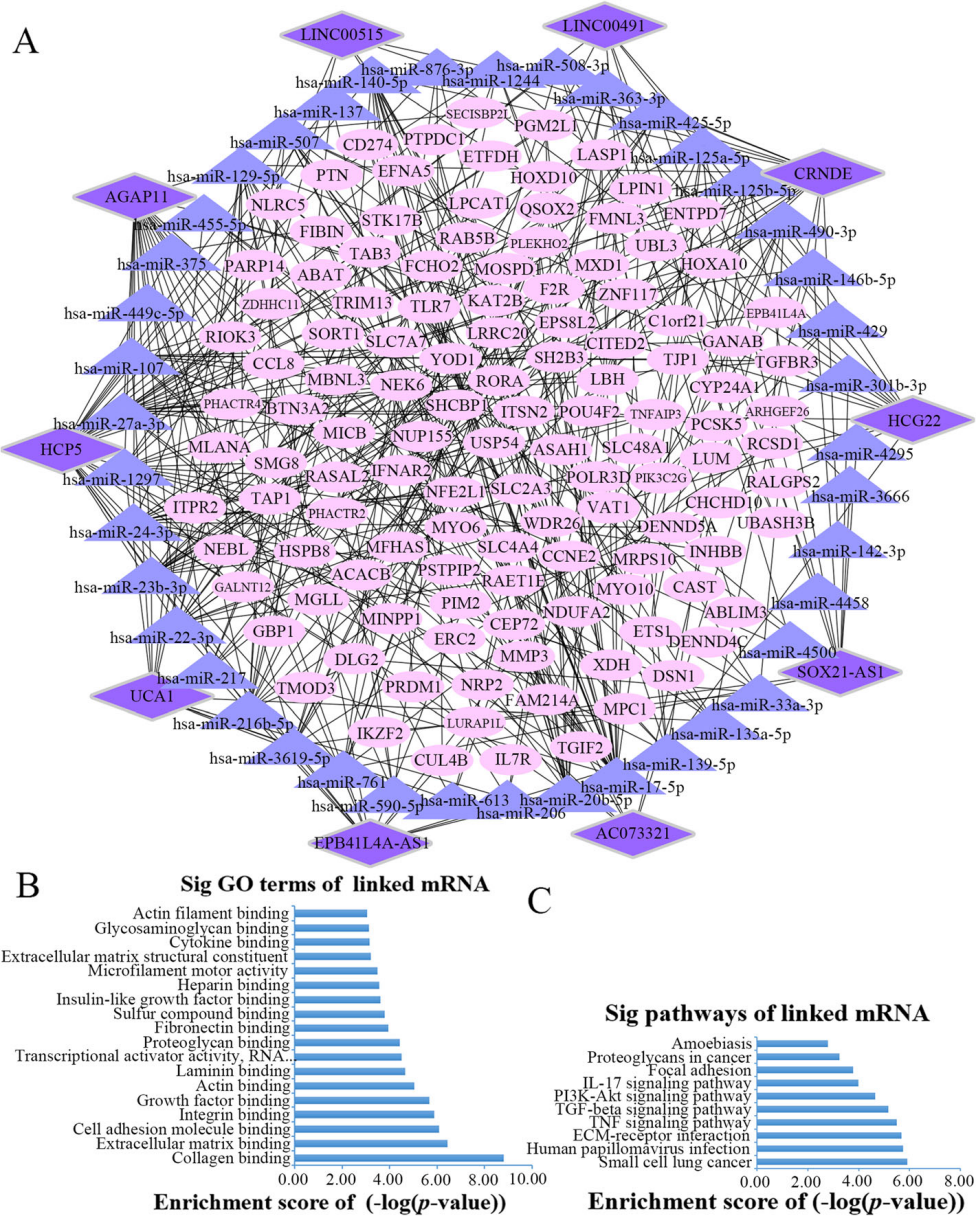
The lncRNA associated ceRNA network and barplots of function enrichment analyses. **a** The lncRNA-miRNA-mRNA ceRNA network. The parallelograms represent lncRNAs, the ellipses represent mRNAs, and the triangles represent miRNAs. **b** The top 18 most significant Gene ontology terms. **c** The top 10 most significant pathway terms

To perform a deeper functional study of the lncRNAs that act as a miRNA sponge in oral cancer, we built a ceRNA network and applied the Cytoscape software to perform visualization (Fig. 4a). The network included 10 lncRNA nodes, 41 miRNA nodes, 122 mRNA nodes and 238 edges. In addition, several miRNAs in the network had been identified in oral cancer as listed in Table 1.

**Table 1:** The effect characteristic of miRNAs in oral squamous cell carcinoma (OSCC)

miRNA	Targets	Effect characteristic	Reference
hsa-miR-139-5p	AKT	Inhibits oral cancer cell proliferation and induces cell apoptosis	[36]
hsa-miR-17-5p	Casp-2/−7/− 8, Bcl-2 and DIALO	Inhibits hypoxia-induced apoptosis	[37]
hsa-miR-107	p53	p53 upregulates miR-107 in normal condition	[38]
hsa-miR-375	Sp1 and cyclin D1	Inhibits cell growth and its expression is correlatedwith prognosis of TSCC	[39]
hsa-miR-455-5p	UBE2B	Binds SMAD3-specific promoter regions, leads toUBE2B downregulation, and contributes to oralcancer tumorigenesis	[40]
hsa-miR-137	CDK6	Arrests cell cycle at the G1-S checkpoint	[41]
hsa-miR-140-5p	ADAM10	Inhibits the invasion and migration of TSCC cells	[42]
hsa-miR-125b-5p	BAK1	Boosts the level of BAK1 that is controlling the apoptotic pathway	[38]

### Functional enrichment analysis

Speculations on the possible functions of the lncRNAs were made through the functional enrichment analysis of their linked mRNAs. GO analysis was performed to analyze the functions of the mRNA nodes, and the GO interaction network was constructed using the BiNGO tool (Additional file 4). As a result, 18 GO terms were found to be significantly enriched (Fig. 4b and Additional file 5). Among these terms, the top three enriched ones were collagen binding, extracellular matrix binding and cell adhesion molecule binding, all of which belonged to the molecular function (MF) terms. Interestingly, extracellular matrix (ECM) binding was related to the proliferation of OSCC cells [43] and played an important role in the growth and survival of oral cancer cells [44]. It was demonstrated that cell adhesion molecules, together with tumor-associated matrix molecules, are functionally involved in the progression of oral cancer [45]. What’s more, discoidin domain receptor-1 (DDR1) could be activated by the specific binding with collagens (II,III) [46], and the activation of DDR1 has been reported in oral cancer [47]. The KEGG pathway analysis resulted in 10 enriched pathway terms, shown in Fig. 4c, including the terms of ECM-receptor interaction, small cell lung cancer, PI3K-Akt signaling pathway, focal adhesion, TGF-β signaling pathway and more (Additional file 6). Among these pathways, ECM-receptor interaction [48], PI3K-Akt signaling pathway [49]**,** TNF signaling pathway [50] and TGF-β signaling pathway [51] were OSCC-related pathways.

### Topological analysis of the ceRNA network

In order to identify the hub genes in the lncRNA-miRNA-mRNA network that are related to oral cancer, we computed the node degrees. In the study of Han et al. [33], the nodes with a degree greater than 5 were defined as hubs. Based on this research, a total of 42 nodes could be chosen as hubs, including 10 lncRNAs, 28 miRNAs and 4 mRNAs (Table 2 and Additional file 7). In addition, BC was also calculated as a measure to select the hubs [52] (Table 3). Higher BC values of the nodes indicated an increased important of these nodes in the regulatory network [53].

**Table 2:** The list of differentially expressed genes (node degree > 5)

Number	Degree	Name	Type
1	27	hsa-miR-17-5p	miRNA
2	24	HCP5	lncRNA
3	21	AGAP11	lncRNA
4	19	hsa-miR-24-3p	miRNA
5	18	hsa-miR-27a-3p	miRNA
6	17	hsa-miR-1297	miRNA
7	17	hsa-miR-23b-3p	miRNA
8	16	hsa-miR-140-5p	miRNA
9	16	hsa-miR-129-5p	miRNA
10	15	CRNDE	lncRNA
11	15	hsa-miR-22-3p	miRNA
12	15	hsa-miR-20b-5p	miRNA
13	14	HCG22	lncRNA
14	13	EPB41L4A-AS1	lncRNA
15	13	hsa-miR-507	miRNA
16	13	hsa-miR-107	miRNA
17	13	hsa-miR-216b-5p	miRNA
18	12	SOX21-AS1	lncRNA
19	12	hsa-miR-3619-5p	miRNA
20	11	UCA1	lncRNA
21	11	hsa-miR-761	miRNA
22	9	hsa-miR-490-3p	miRNA
23	9	hsa-miR-125b-5p	miRNA
24	9	hsa-miR-1244	miRNA
25	9	hsa-miR-139-5p	miRNA
26	7	YOD1	mRNA
27	7	LINC00491	lncRNA
28	7	hsa-miR-125a-5p	miRNA
29	7	hsa-miR-425-5p	miRNA
30	7	hsa-miR-363-3p	miRNA
31	7	hsa-miR-876-3p	miRNA
32	7	hsa-miR-455-5p	miRNA
33	7	hsa-miR-217	miRNA
34	6	NEK6	mRNA
35	6	MFHAS1	mRNA
36	6	RORA	mRNA
37	6	hsa-miR-33a-3p	miRNA
38	6	LINC00515	lncRNA
39	6	AC073321	lncRNA
40	6	hsa-miR-146b-5p	miRNA
41	6	hsa-miR-508-3p	miRNA
42	6	hsa-miR-135a-5p	miRNA

**Table 3:** List of the 15 genes with the top betweenness centrality

Number	Type	name	Betweenness Centrality
1	lncRNA	HCP5	0.26
2	lncRNA	AGAP11	0.16
3	miRNA	hsa-miR-17-5p	0.14
4	miRNA	hsa-miR-24-3p	0.13
5	miRNA	hsa-miR-27a-3p	0.10
6	miRNA	hsa-miR-1297	0.10
7	miRNA	hsa-miR-22-3p	0.09
8	lncRNA	CRNDE	0.09
9	miRNA	hsa-miR-140-5p	0.09
10	miRNA	hsa-miR-129-5p	0.08
11	lncRNA	HCG22	0.08
12	miRNA	hsa-miR-216b-5p	0.07
13	miRNA	hsa-miR-507	0.07
14	miRNA	hsa-miR-23b-3p	0.06
15	miRNA	hsa-miR-107	0.06

We found that the three lncRNAs of HCP5, AGAP11 and HCG22 had higher node degrees along with greater BC values, suggesting that they may be potential key regulators controlling the oral cancer related ceRNA network.

### Key lncRNA-miRNA-mRNA subnetwork

**Fig. 5:**
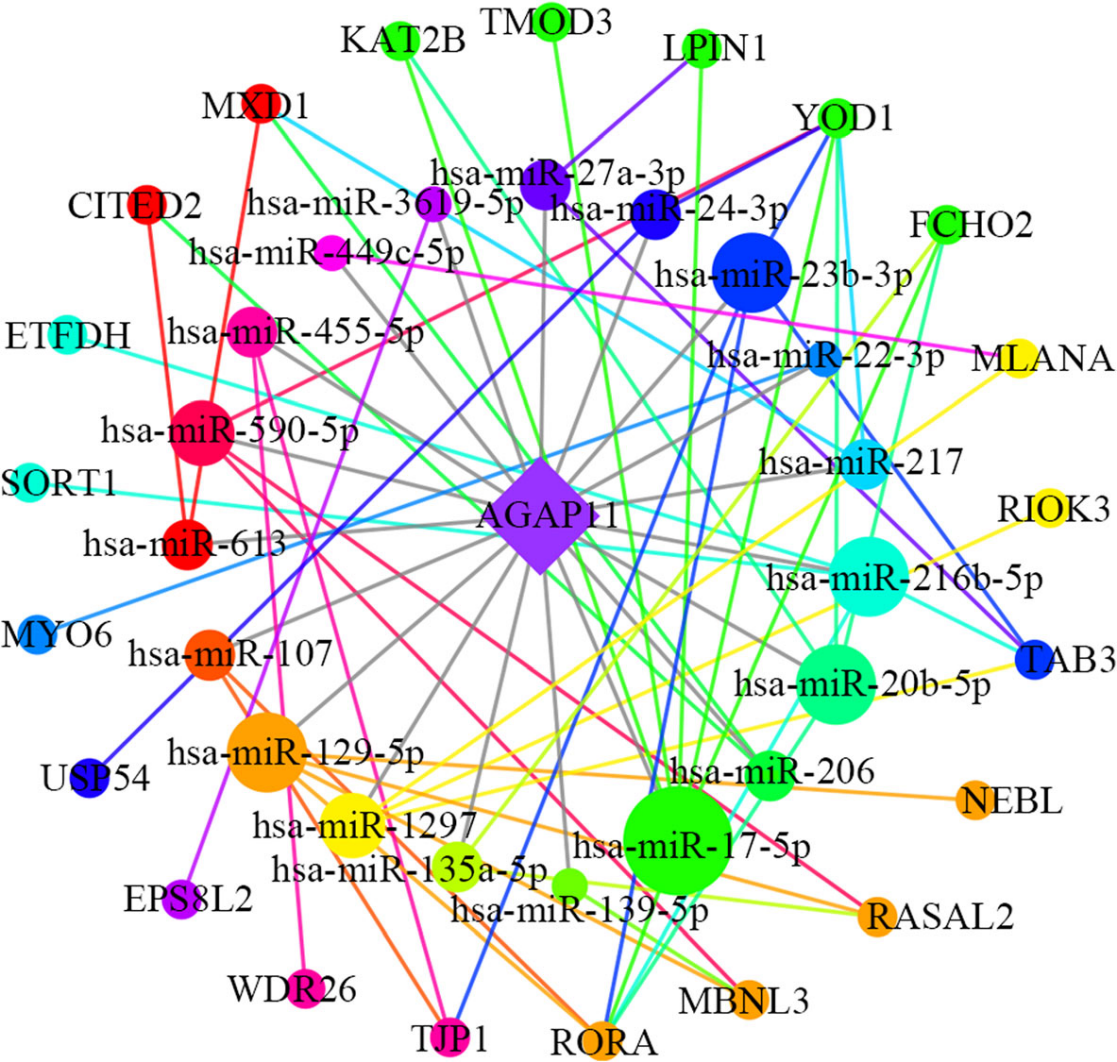
The sub-network of hub lncRNA AGAP11. The rhombuses represent lncRNAs, the circles on the inner loop represent miRNAs, and the circles on the outer loop represent mRNAs. The bigger size circles have, the more nodes they are connected to

**Fig. 6:**
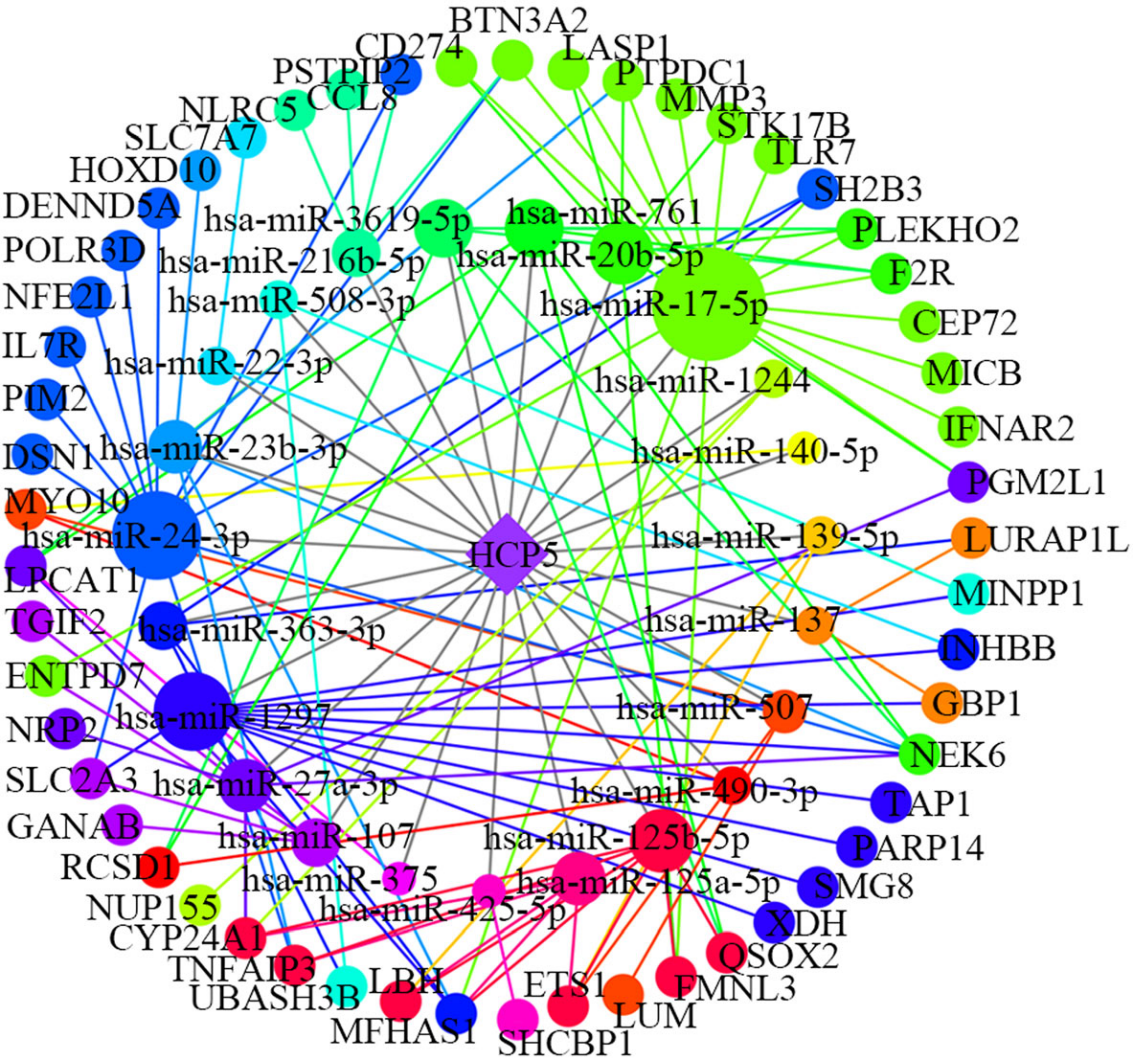
The sub-network of hub lncRNA HCP5. The rhombuses represent lncRNAs, the circles on the inner loop represent miRNAs, and the circles on the outer loop represent mRNAs. The bigger size circles have, the more nodes they are connected to

**Fig. 7:**
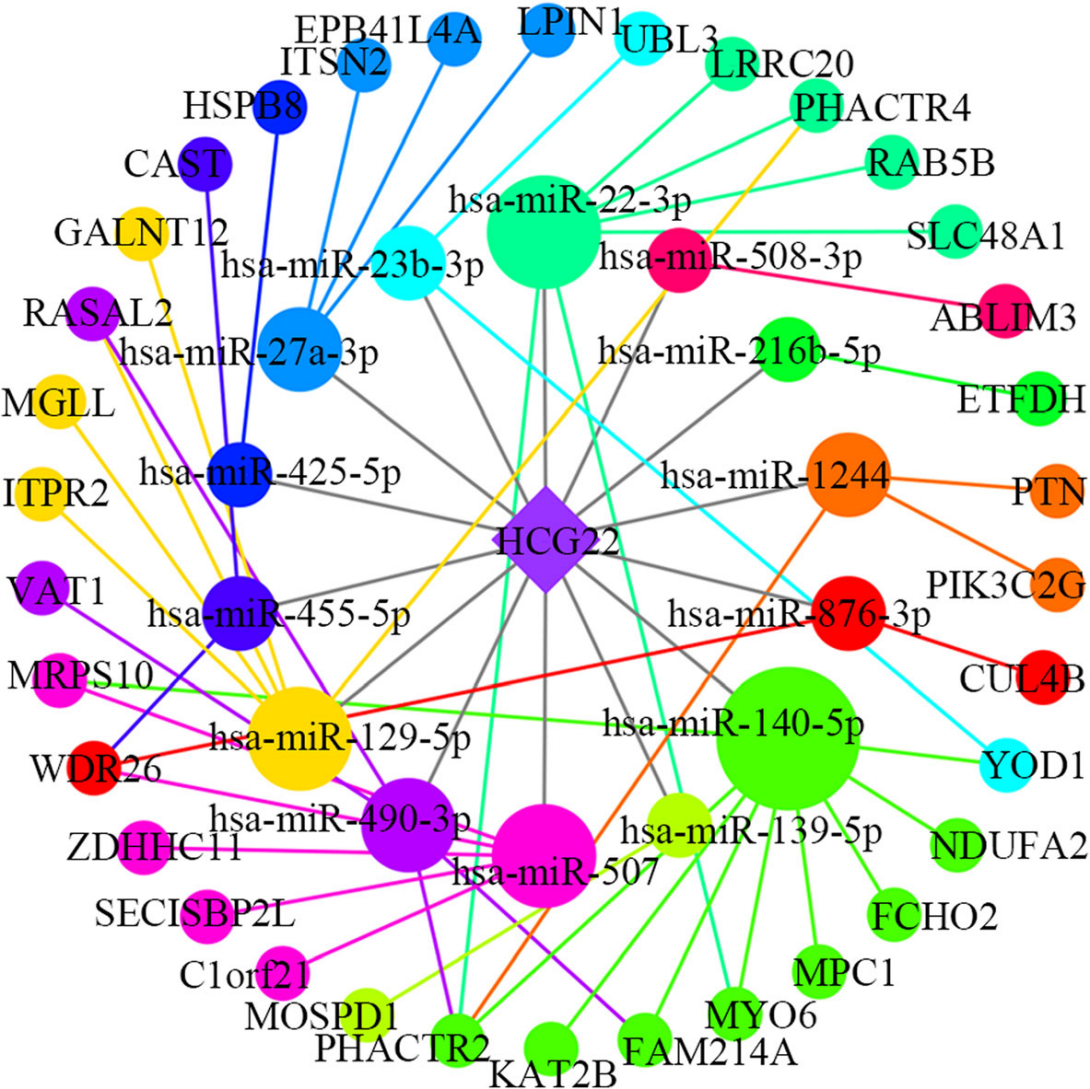
The sub-network of hub lncRNA HCG22. The rhombuses represent lncRNAs, the circles on the inner loop represent miRNAs, and the circles on the outer loop represent mRNAs. The bigger size circles have, the more nodes they are connected to

Based on the above-conducted analysis, we obtained three key lncRNAs: HCP5, AGAP11 and HCG22, which may play a role in oral cancer. These hub lncRNAs were used with their linked miRNAs and mRNAs to construct three more specific functional lncRNA-associated subnetworks. The AGAP11-associated subnetwork included 1 lncRNA, 19 miRNAs, 21 mRNAs and 48 edges (Fig. 5). As shown in Fig. 6, the subnetwork of HCP5 consisted of 1 lncRNA, 23 miRNAs, 53 mRNAs and 110 edges. As for HCG22, it interacted with 14 miRNAs and 34 mRNAs (Fig. 7).

**Fig. 8:**
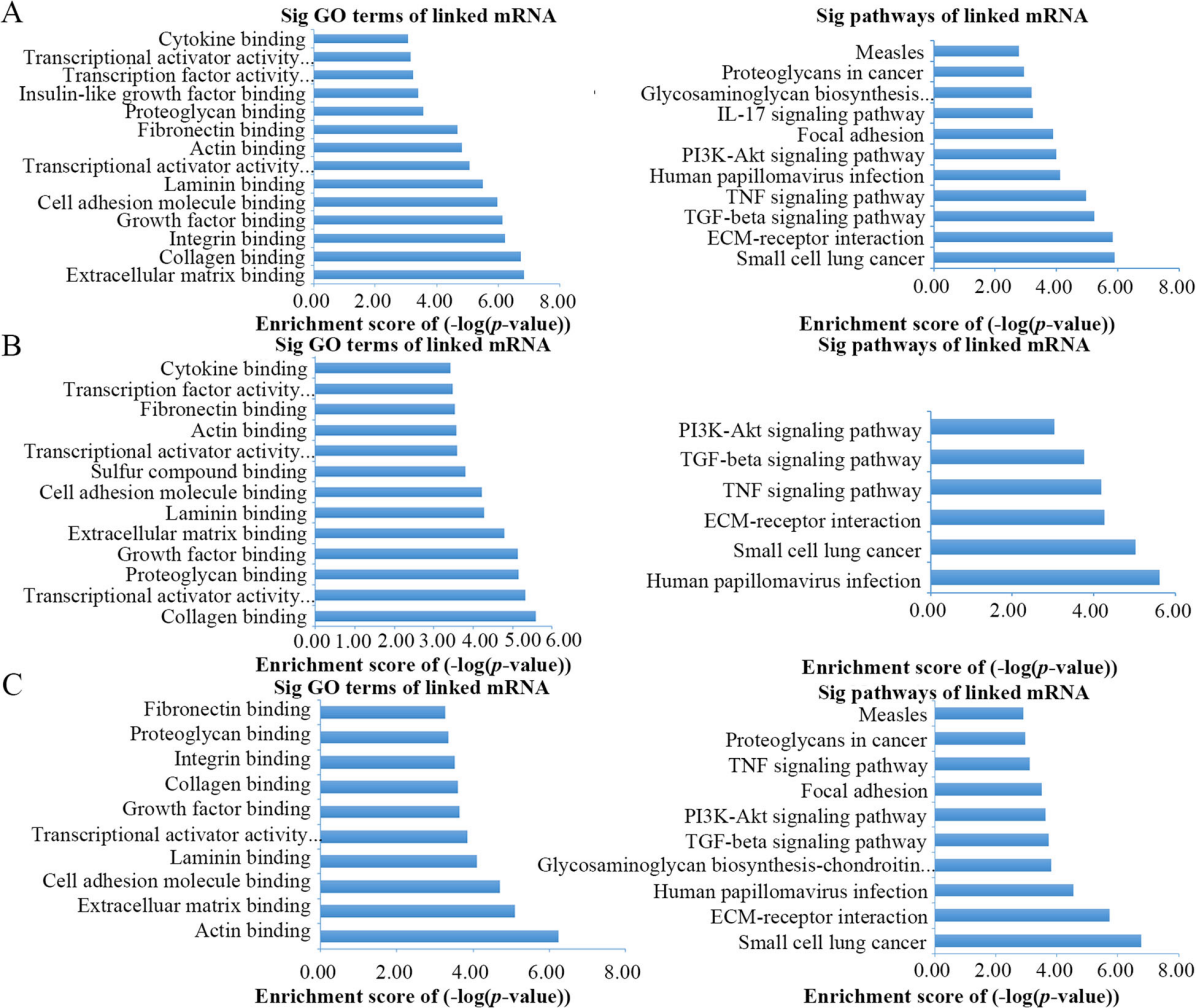
The barplots of function enrichment analyses. **a** Functional enrichment analyses for AGAP11-related mRNAs. **b** Functional enrichment analyses for HCP5-related mRNAs. **c** Functional enrichment analyses for HCG22-related mRNAs

To further understand the biological functions of the three hub lncRNAs, we performed GO functional enrichment analysis and KEGG pathway analysis for each hub-associated subnetwork. The results of the functional enrichment analysis revealed 14 enriched GO terms and 11 enriched pathway terms in the AGAP11-associated subnetwork (Fig. 8a**)**, while there were 15 enriched GO terms and 6 enriched KEGG terms in the HCP5-associated subnetwork (Fig. 8b**)**. Regarding the HCG22-associated subnetwork***,*** there were 10 differentially enriched GO terms and 10 enriched KEGG pathways (Fig. 8c).

### Differentially expressed RNAs in TCGA data

A total of 236 oral cancer samples and 44 normal samples were obtained after data preprocessing. To enhance the data reliability, genes with high expression values (data values were more than the third quartile) were filtered for further analysis. However, lncRNA AGAP11 was excluded. We identified 193 differentially expressed RNAs: 99 genes were downregulated and 94 were upregulated. The full list of differentially expressed RNAs was shown in Additional file 8.

### Functional enrichment analysis

GO analysis of the up-regulated genes revealed that 17 enriched clusters were associated with biological processes (BP), 6 with cellular components (CC), and 9 with molecular function (MF) (Additional file 9). Among these terms, the top three enriched biological process were cell-cell adhesion, extracellular region and water transporter activity. Functional enrichment analysis of the down-regulated genes showed that 84 enriched clusters were associated with BP, 5 with CC, and 9 with MF (Additional file 10). Among them, 26 genes were enriched in sequence-specific DNA binding and represented the lowest FDR.

### Survival analysis

**Fig. 9:**
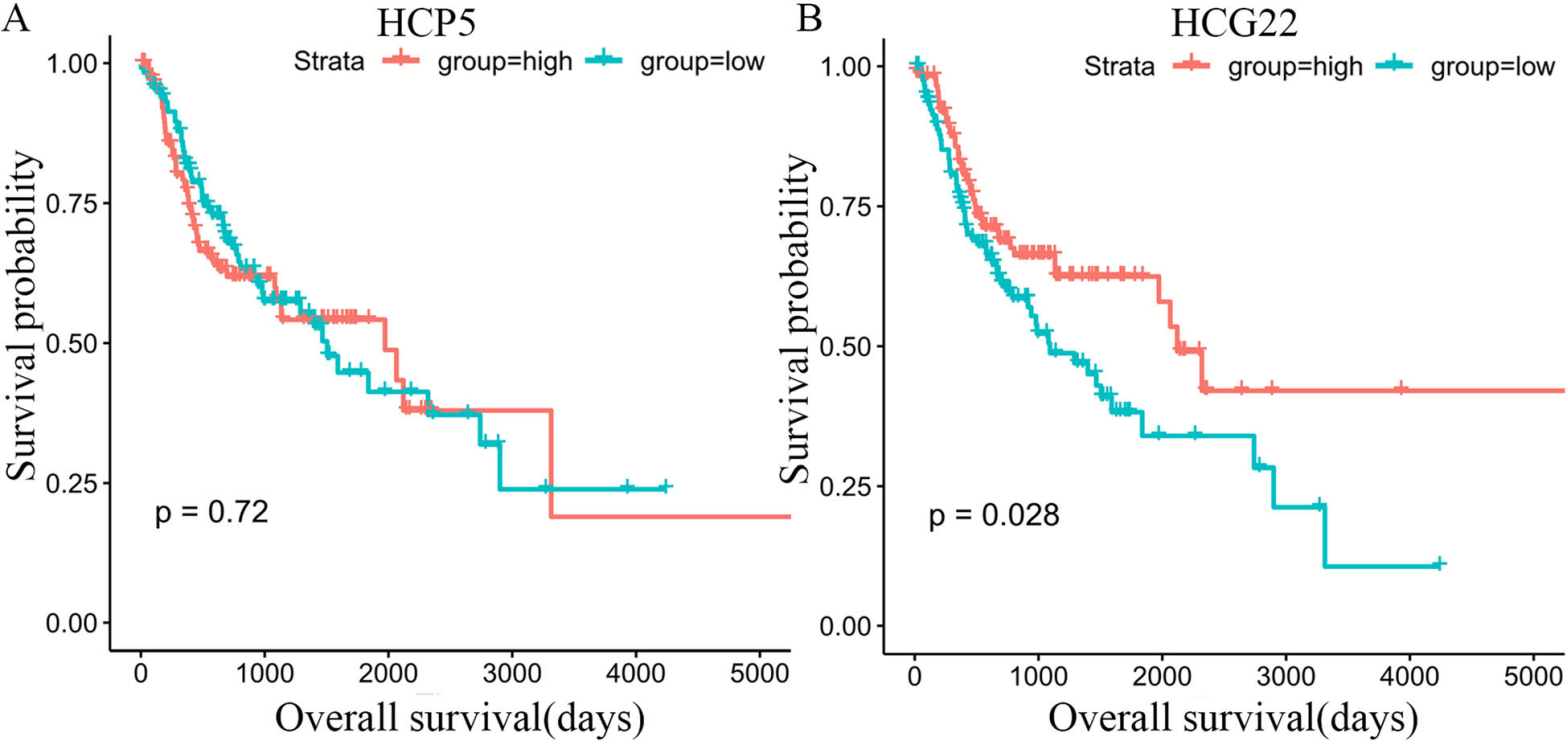
Survival curves for the hub lncRNAs associated with overall survival (OS). **a** HCP5 showed no significant correlation with OS in oral cancer. **b** HCG22 was also significantly associated with survival(*p* < 0.05). The horizontal axis represents overall survival time (days), vertical axis represents survival function

Survival analysis was estimated based on Kaplan-Meier curve analysis. The difference was statistically significant with log-rank *P* < 0.05. As a result, HCG22 was significantly correlated with reduced survival time in patients with oral cancer (Fig. 9b), while HCP5 showed no significant correlation with overall survival in oral cancer (Fig. 9a).

## Discussion

In this study, we used the expression profiles from the NCBI GEO to construct a lncRNA-associated network of oral cancer based on the ceRNA theory. According to the results of the bioinformatics prediction and correlation analyses, the nodes of the network included 10 lncRNAs, 41 miRNAs and 122 mRNAs. Functional enrichment analysis was performed to reveal the biological functions of the mRNAs, the ceRNA counterparts of lncRNA [53]. As a result, three differentially enrichment GO terms (collagen binding [54], extracellular matrix binding [43] and cell adhesion molecule binding [45]) were associated with oral cancer. Moreover, four KEGG pathways (TGF-β signaling pathway [51], ECM-receptor interaction [44], TNF-signaling pathway [50] and PI3K-Akt signaling pathway [49]) showed to be related to the tumorigenesis of OSCC. It was shown that the nodes with a higher degree of connectivity to other nodes are often more important in the network. By applying the topology analysis to the ceRNA network, we found three lncRNAs (HCP5, AGAP11 and HCG22) with significantly higher degrees and BC values compared with the other nodes, which means that these hubs are essential in the network organization and play a critical role in the ceRNA network. However, the average expression level of AGAP11 in oral cancer samples obtained from TCGA database was low. Subsequently, the prognostic value of each hub lncRNA was evaluated using Kaplan-Meier curve and Log-rank method. We found that HCG22 was positively correlated with overall survival and considered HCG22 as a key lncRNA responsible for the prognosis of oral cancer. Therefore, we supposed that HCG22 may play a more significant role in the pathogenesis and prognosis of oral cancer.

HCG22 (HLA complex group 22) is a long non-coding RNA gene that has been found to be down-regulated in oral cancer recently [55]. Low-expression of HCG22 has been confirmed to be associated with several types of diseases, including esophageal squamous cell carcinoma [56], bladder cancer [57], and steroid-induced ocular hypertension [58]. Consistent with another previous study [59], we identified that the low expression level of lncRNA HCG22 was associated with poor survival in oral cancer. However, there is no experimental evidence to support the contribution of HCG22 to the development of oral cancer. Based on the HCG22-associated subnetwork, we proposed that HCG22 might be an essential regulator in oral cancer by being a sponge for miRNAs. Several miRNAs competed by HCG22 and mRNAs were associated with oral cancer. For example, miR-139-5p could induce oral cancer cell apoptosis through the Akt signaling pathway [36] and could be used as an effective biomarker to detect the tongue squamous cell carcinoma (TSCC) [60]. Another study has demonstrated that miR-140-5p targeted ADAM10 and inhibited the invasion and migration of the TSCC cells [42]. These studies may support our proposal of the regulatory function of HCG22 in oral cancer. Moreover, functional annotations of the 34 putative target mRNAs in the HCG22-miRNA-mRNA subnetwork revealed the biological functions of HCG22. It has been demonstrated that normal cell migration requires interactions with the extracellular matrix (ECM), which mainly includes collagens, laminins and fibronectin [44], while changes in the composition of ECM may contribute to the development and invasion of the oral cancer cells. In detail, it was observed that more fibrillary collagen type III than thick collagen type I existed in poorly-differentiated SCC compared with well-differentiated one [61]. When oral cancer cells invaded the connective tissue region from the basal membrane, a switch in the ECM’s composition from a laminin-enriched environment to a collagen and fibronectin-enriched one would influence the metastatic and invasive behavior of the tumor cells, since the tumor formation was highly sensitive to the microenvironment [62]. Another GO term, regulation of transcription by RNA polymerase II, also had a connection with oral cancer. Xu et al. provided evidence that Histone acetylation and RNA polymerase II recruitment on the integrin β6 promoter are involved in the TGF-β1-induced integrin β6 expression in OSCC cells, which would then promote tumorigenesis and metastasis [63]. Additionally, ten KEGG pathways, including the human papillomavirus infection, ECM-receptor interaction, small cell lung cancer, TGF-β signaling pathway, PI3K-Akt signaling pathway and TNF signaling pathway were determined. In accordance with the results from a previous report, the ECM-receptor interaction pathway was one of the most significantly altered pathways in the OSCC samples [64]. Tang et al. [50] indicated that TNF-α enhances the invasion and metastasis ability of the OSCC cells via the NF-Kb signaling pathway. The TGF-β/Smad pathway contributed to oral cancer tumorigenesis [51], and the PI3K-Akt signaling pathway was also considered to be important in the development of OSCC [49]. In summary, HCG22 may have the potential to become a novel biomarker for the detection and diagnosis of oral cancer.

Importantly, these results provided us with important information regarding the diagnostic and prognostic role of lncRNAs in oral cancer and pointed out lncRNA HCG22 as a candidate prognosis biomarker or potential therapeutic target. However, since no verification experiments were included in our study, the functional role of HCG22 still needs further investigation.

## Conclusion

Overall, we constructed a lncRNA–miRNA–mRNA network based on the ceRNA theory, which enabled us to screen and analyze the lncRNAs that play functional roles in the progression of OSCC as miRNA sponges. Furthermore, we identified a hub lncRNA HCG22 in the complex ceRNA network. This study offered a unique insight into the ceRNA regulation network in OSCC and laid the foundation for further experimental and clinical research.

## Supplementary information

**Additional file 1.** Data banks/repositories corresponding to all datasets analyzed in this study.

**Additional file 2.** TCGA datasets of all oral cancer samples obtained from the TCGA. The data included 305 oral cancer samples (14 samples of non-solid tumors were excluded) and 44 matched normal samples.

**Additional file 3.** Potential co-expression competing triples (lncRNA-miRNA-mRNA triples)

**Additional file 4. **Gene ontology (GO) terms interaction network. Yellow nodes mean nodes with *P*-value < 0.05 and Benjamini corrected *P*-value < 0.05.

**Additional file 5.** The enriched Gene ontology (GO) terms of linked mRNA in the ceRNA network.

**Additional file 6.** The enriched pathway terms of linked mRNA in the ceRNA network.

**Additional file 7.** All node degree analysis reveals the distribution of the points with different node degrees in ceRNA network.

**Additional file 8.** All differentially expressed genes identified between oral cancer tissues and matched normal tissues.

**Additional file 9.** The enriched Gene ontology (GO) terms of up-regulated genes in oral cancer.

**Additional file 10.** The enriched Gene ontology (GO) terms of down-regulated genes in oral cancer.

## Data Availability

The datasets supporting the conclusions of this article were retrieved from the GEO repository (https://www.ncbi.nlm.nih.gov/geo/) with the accession number GSE74530 and the series matrix files were available at (ftp://ftp.ncbi.nlm.nih.gov/geo/series/GSE74nnn/GSE74530/matrix/). The raw data for data quality assessment was available at (https://www.ncbi.nlm.nih.gov/geo/download/?acc=GSE74530&format=file). The full data table of microarray platform GPL570 was available at (https://www.ncbi.nlm.nih.gov/geo/query/acc.cgi?acc=GPL570). The human comprehensive gene annotation we used to identify lncRNAs was available at (ftp://ftp.ebi.ac.uk/pub/databases/gencode/Gencode_human/release_34/gencode.v34.annotation.gtf.gz). The case IDs and direct web links of all oral cancer samples obtained from the TCGA data portal (http://portal.gdc.cancer.gov/; dbGaP Study Accession: phs000178) by Bioconductor package TCGAbiolinks were shown in Additional file 2. Data banks/repositories corresponding to all datasets analyzed in this study were listed in Additional file 1.

## Abbreviations

BC: Betweenness centrality
BiNGO: Biological networks gene ontology tool
ceRNA: Competitive endogenous RNA
DEG: Differentially expressed gene
DEL: Differentially expressed lncRNA
DEM: Differentially expressed mRNA
ECM: Extracellular matrix
GEO: Gene expression omnibus database
GO: Gene ontology
KEGG: Kyoto encyclopedia of genes and genomes
RLE: Relative log expression
lncRNA: Long non-coding RNA
miRNA: microRNA
mRNA: Messenger RNAs
NCBI: National center for biotechnology information
ncRNA: Non-coding RNA
OSCC: Oral squamous cell carcinoma
PCC: Pearson’s correlation coefficient
TCGA: The cancer genome atlas
