# Supplementary figures and images for: Construction and analysis of a lncRNA-miRNA-mRNA network based on competitive endogenous RNA reveal functional lncRNAs in oral cancer
Source: BMC Med Genomics. 2020 Jun 22;13:84. doi: 10.1186/s12920-020-00741-w (PMC7310129; doi:10.1186/s12920-020-00741-w)

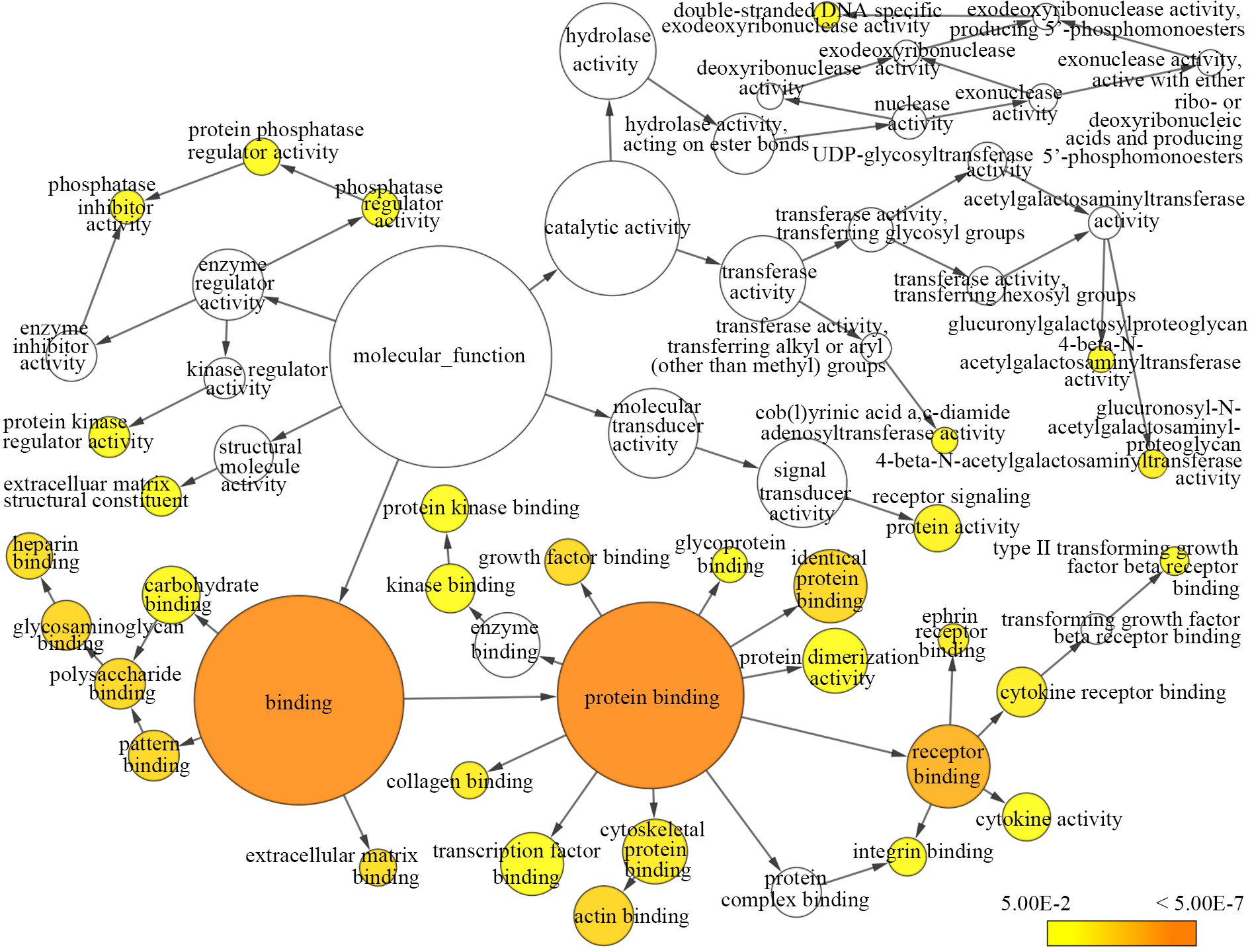

Supplement: Supplementary file 4 — Additional file 4. Gene ontology (GO) terms interaction network. Yellow nodes mean nodes with P-value < 0.05 and Benjamini corrected P-value < 0.05. [file 12920_2020_741_MOESM4_ESM.tiff]

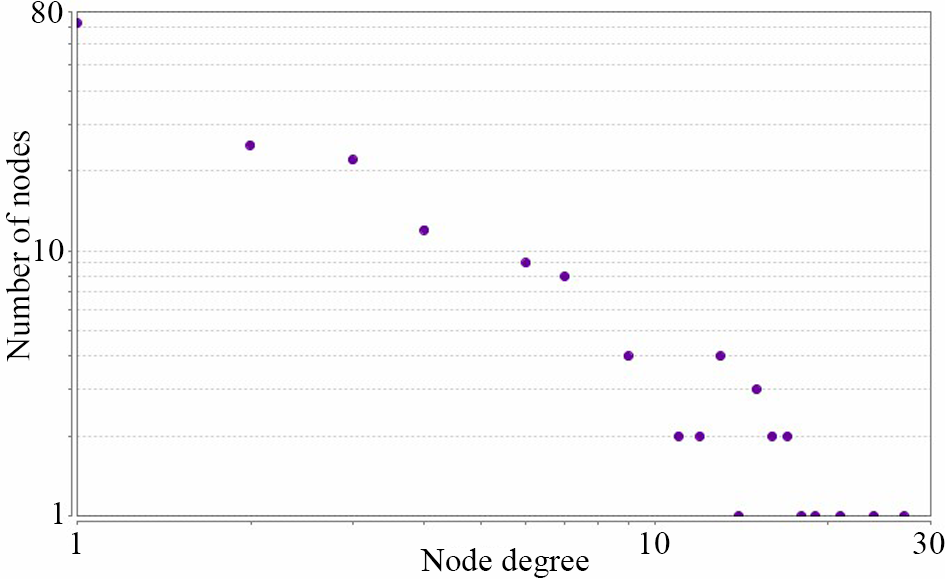

Supplement: Supplementary file 7 — Additional file 7. All node degree analysis reveals the distribution of the points with different node degrees in ceRNA network. [file 12920_2020_741_MOESM7_ESM.tiff]
